# A Rare Association Between Gastrointestinal Stromal Tumor and Neurofibromatosis Type 1: A Case Report

**DOI:** 10.7759/cureus.34148

**Published:** 2023-01-24

**Authors:** Mohammad Abu-Abaa, Ali Abdulsahib, Salman Kananeh, Alaa Aldookhi

**Affiliations:** 1 Internal Medicine, Capital Health Regional Medical Center, Trenton, USA

**Keywords:** neurofibroma, tumor, gastrointestinal stromal tumor (gist), obscure gi bleeding, neurofibromatosis

## Abstract

The association between gastrointestinal stromal tumor (GIST) and neurofibromatosis type 1 (NF1) has been documented in medical literature but remains rare. We report a 53-year-old male patient who was investigated extensively for months for lower gastrointestinal tract (GIT) bleeding that remained obscure despite upper and lower endoscopies as well as a barium follow-through. His past medical history is significant for NF1 with numerous cutaneous neurofibromas as well as café au lait spots and bilateral functional pheochromocytoma status post-bilateral adrenalectomy. However, the progression of his bleeding as well as iron deficiency anemia prompted more aggressive investigations. These have revealed a small bowel mass that proved to be GIST on histological and immunohistochemical staining examination. This case helps remind clinicians of the important association between NF1 and GIST, and the clinical pearl that most GISTs in NF1 are located in the small intestine and may not be apparent on endoscopy with barium follow-through and require push enteroscopy to allow for better localization.

## Introduction

A gastrointestinal stromal tumor (GIST) is a rare tumor with high malignancy potential [1]. Progression to malignancy happens in 30% of cases [2]. They are estimated to account for 1%-2% of all gastrointestinal tumors, although the true incidence is unknown as a significant proportion of the tumors are asymptomatic [3]. They can affect the entire gastrointestinal tract (GIT). They are usually misdiagnosed as leiomyoma or leiomyosarcoma [4]. These tumors arise from the interstitial cells of Cajal, which are responsible for GIT motility [2]. Of all GISTs, 85% have a sporadic activating mutation of the KIT gene, and the rest have a mutation of the platelet-derived growth factor receptor alpha (PDGFRA) gene, both of which encode for tyrosine kinase. This leads to hyperfunction of tyrosine kinase, and therefore, tyrosine kinase inhibitors are the mainstay of therapy with/without surgical resection [5]. Chemotherapy and radiation are not usually effective [1].

## Case presentation

A 53-year-old male patient presented to the emergency department (ED) for worsening intermittent melena for three months. During this period, the source of bleeding was not revealed by both esophagogastroduodenoscopy (EGD) and colonoscopy. Small bowel follow-through was also negative. EGD showed only erythematous gastric mucosa, and the patient was started on proton pump inhibitors. Colonoscopy showed non-bleeding hemorrhoids and two rectal hyperplastic polyps that were removed. His past medical history is remarkable for neurofibromatosis type 1 (NF1), bilateral functional pheochromocytoma status post-bilateral adrenalectomy, primary progressive multiple sclerosis, and paroxysmal atrial fibrillation without anticoagulation. He was on long-term glucocorticoids and mineralocorticoid replacement therapy. His family history was remarkable for multiple sclerosis in multiple family members as well as neurofibromatosis type 1 in his father. He was asymptomatic otherwise. In the ED, vitals signs included a temperature of 37.2°C, heart rate of 97 beats per minute, respiratory rate of 19 cycles per minute, blood pressure of 105/75 mmHg, and SpO2 of 96% on room air. His physical examination showed widespread cutaneous neurofibromas and a soft, non-tender, and non-distended abdomen with normal bowel sounds. Conjunctival pallor was evident. His rectal examination was negative with negative guaiac. Otherwise, his physical examination was unremarkable. Basic laboratory results showed hemoglobin of 7.1 g/dL, which has dropped from 9.3 g/dL nine days prior, with mean corpuscular volume (MCV) of 71.2 fL and red cell distribution width (RDW) of 15.7% (reference: 11.6%-15.4%). No evidence of leukopenia nor thrombocytopenia was seen. Iron studies showed low serum iron at 17 mcg/dL (reference: 49-181 mcg/dL), low ferritin at 5.16 ng/mL (reference: 17.9-464 ng/mL), low iron saturation at 5% (reference 20%-55%), and low total iron-binding capacity at 250 mcg/dL (reference: 261-462 mcg/dL) (Table 1). He tested positive for COVID-19 infection. Otherwise, his laboratory findings were unremarkable. Iron infusion therapy was started.

**Table 1:** Laboratory findings The table shows the patient’s laboratory findings prior to, during, and after admission. MCV: mean corpuscular volume, RDW-CV: red cell distribution width-coefficient of variation

Variable	Baseline four months prior to admission	Three months prior to admission	On admission	One-month follow-up	Reference
White blood cell count	10,000 cells/mL	14,000 cells/mL	8,900 cells/mL	4,800 cells/mL	4,000-10,000 cells/mL
Hemoglobin	13.7 g/dL	12.8 g/dL	7.1 g/dL	8.7 g/dL	13.7-17.5 g/dL
MCV	86.6 fL	86.3 fL	71.2 fL	76.7 fL	79-95 fL
RDW-CV	13.6%	14.8%	15.9%	21.9%	11.6%-15.4%
Platelet	220	233	275	298	150-400
Serum iron	-	-	17 mcg/dL	-	49-181 mcg/dL
Ferritin	-	-	5.16 ng/mL	-	17.9-464 ng/mL
Iron saturation	-	-	5%	-	20%-55%
Total iron-binding capacity	-	-	250 mcg/dL	-	261-462 mcg/dL

**Figure 1:**
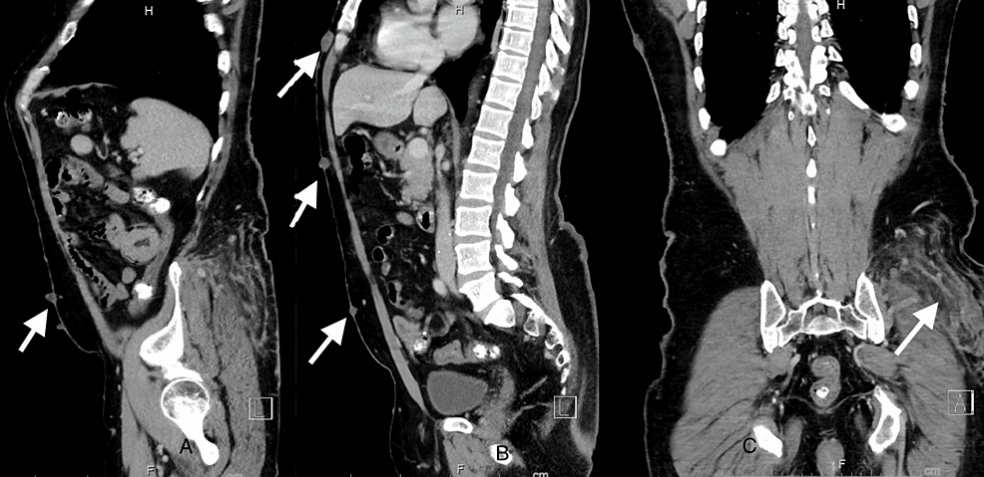
CT scan of the abdomen Arrows in A and B images show cutaneous neurofibromas. The arrow in the C image shows complex neurofibroma. CT: computed tomography

**Figure 2:**
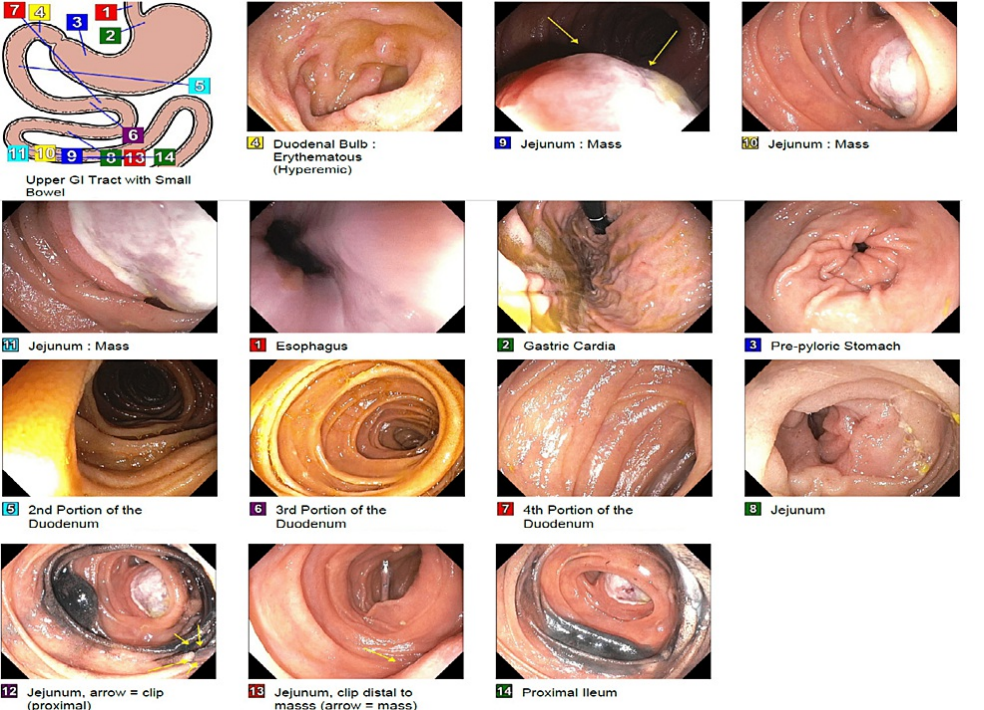
EGD EGD with push enteroscopy shows a jejunal ulcerated mass (yellow arrows in images 9, 12, and 13). EGD: esophagogastroduodenoscopy

Computed tomography (CT) angiography of the abdomen and pelvis with contrast was unremarkable, except for multiple soft tissue nodules along the skin compatible with cutaneous neurofibromas as well as nodular soft tissue infiltrating left gluteal soft tissue compatible with plexiform neurofibroma (Figure 1). A repeat EGD with push enteroscopy was pursued that revealed a large polypoid and ulcerated mass with stigmata of recent bleeding in the jejunum about 60 distal to the pylorus that was deemed not amenable for endoscopic therapy (Figure 2). His corticosteroids were switched to intravenous form, and the patient underwent robotic small bowel resection. Pathological examination showed a 2-cm gastrointestinal stromal tumor (GIST) of spindle cell type and low grade, involving the submucosa, muscularis propria subserosa, and serosa, with benign lymph nodes. Tumor cells were positive for discovered on GIST-1 (DOG1). Hemoglobin remained stable during the period of hospitalization, and he did not require a blood transfusion.

A follow-up one month later revealed no further clinical evidence of bleeding and hemoglobin improvement to 8.7 g/dL.

## Discussion

GISTs are usually asymptomatic. The median age is 65 years, and the small bowel is the second most common location in 32% of cases after the stomach in 56% of cases [4]. It is seen equally in men and women [6]. Tumors located outside the stomach tend to have a higher malignancy potential. The most common site of metastasis is the liver, and lung and bone metastases have also been reported [2]. It has three histological types: spindle cell in 70% of cases, epithelioid in 20% of cases, and mixed in 10% of cases [4]. Around 18% of cases are asymptomatic and discovered incidentally. Nonspecific symptoms of nausea, early satiety, weight loss, anorexia, dyspepsia, fever, constipation, and abdominal discomfort can be present. Exophytic tumors can also present with symptoms of compression of surrounding structures such as jaundice and dysphagia. Intraluminal tumors can lead to intestinal obstruction. Bleeding is usually the result of pressure necrosis and ulceration [7].

The diagnostic test of choice is CT enterography, while magnetic resonance imaging (MRI) is best used to detect hepatic metastases as well as characterize rectal tumors [8]. GIST also can undergo necrosis and calcification, which becomes visible on CT scans [9]. Exophytic tumors are more common than intraluminal tumors, and the classical appearance of intraluminal tumors is a punched-out ulcer [10]. However, the ultimate diagnosis is based on histological and immunohistochemical staining and examination. Recent reports indicate that DOG1 is more sensitive and specific than cluster of differentiation 117 (CD117). This sensitivity is much less in those with PDGFRA mutations from 95% to only 9% [11].

Neurofibromatosis type 1 (NF1) is an autosomal dominant disease resulting from the mutation of the NF1 gene on chromosome 17, which encodes for a tumor suppressor protein called neurofibromin. This results in unregulated cell proliferation and an increased tendency to develop both benign and malignant tumors [12]. GIT manifestations of NF1 range from neurogenic tumors to neuroendocrine tumors, to stromal tumors. The most common GIT tumor is GIST [13]. The risk of GIST in NF1 is 150-fold higher than in the general population [14].

A retrospective study of 15 cases of neurofibromatosis type 1 (NF1)-associated GIST concluded that it is phenotypically and genotypically distinct from adult sporadic GIST [15]. It is more commonly located in the small intestine, has lower malignant potential, and usually lacks PDGFRA and KIT gene mutations [15]. This study also showed that NF1-associated GIST is more likely to be multifocal and has higher CD34 expression as compared to sporadic GIST [15]. These findings were also replicated in a more recent study of a similar number of NF1-associated GIST [16]. This study also showed that these tumors are mostly of spindle cell type with female predominance and lack of durable response to tyrosine kinase inhibitors [16]. In the absence of KIT and PDGFRA mutations, it is believed that the somatic NF1 mutation itself leads to the overexpression of KIT and/or PDGFRA genes [17].

## Conclusions

GIST is the most common GIT tumor associated with NF1. It should always be considered in the differential diagnosis of GIT pathology/presentation in those with NF1. It is usually asymptomatic but can present with GIT bleeding. NF1-associated GIST differs from sporadic one but lacks known genetic mutations, multifocality, and durable response to tyrosine kinase inhibitors; therefore, surgery is the mainstay of treatment. Unlike sporadic GIST, which most commonly occurs in the stomach, the small intestine is the most common location of GIST in NF1 patients. Localizing the tumor with EGD, colonoscopy, and barium follow-through may be challenging and require push enteroscopy.
